# Editorial: The practical implication of clinical pharmacokinetics in drug development, pharmaceutical analysis, and clinical research

**DOI:** 10.3389/fphar.2023.1252030

**Published:** 2023-07-25

**Authors:** Abad Khan, Lateef Ahmad, Faisal Raza, Saeed Ahmad Khan, Tahir Ali

**Affiliations:** ^1^ Department of Pharmacy, University of Swabi, Swabi, Pakistan; ^2^ School of Pharmacy, Shanghai Jiao Tong University, Shanghai, China; ^3^ Department of Pharmacy, Kohat University of Science and Technology, Kohat, Pakistan; ^4^ Department of Comparative Biology and Experimental Medicine, University of Calgary (UofC), Calgary, NW, Canada

**Keywords:** practical implication, clinical pharmacokinetics, drug development, pharmaceutical analysis, clinical research, bioavailabiliry

In drug discovery, pharmacokinetics (PK) and pharmacodynamics (PD) models are used for identification and selection of a drug candidate, and prediction of clinical response. These models can also facilitate characterizing the mechanism of action and disease behaviour of a given drug aiding in assessing efficacy and safety. Clinical pharmacokinetics aims to predict the best dosage and dosing regimen to ensure and maintain therapeutically effective concentrations at the site of action. Outcomes of clinical pharmacokinetic studies are useful for the rational use of medicines according to the need of patients, and for the prediction of potential drug-interactions.

After successful initial screening of a molecule for a disease, the molecule undergoes evaluation tests and formulation trials to become a new beneficial product. The response of a drug molecule to different populations may vary due to many factors. Pharmacokinetic studies are conducted in different populations to observe any variation in the pharmacokinetic parameters. Pharmacometrics is necessary to achieve all the pharmacokinetic information regarding the new drug. The dosage regimen can be decided from the pharmacokinetic studies and this becomes more critical when narrow therapeutic index drugs are to be administered. Metabolites may have some effects and the rate of metabolite formation is also important in the case of a prodrug. The enzymes responsible (pharmacogenomics) also vary greatly from population to population. An appropriate and important method for the analysis of the drug and metabolite(s) is to quantify them in biological samples, especially human blood samples. The data obtained can be easily evaluated and pharmacokinetic parameters can be accessed. The sensitivity of the analytical method is a major concern, as the developed method must be able to analyze the lowest concentration in the biological matrix. Validation and optimization of a new analytical method are also necessary for accurate quantification of drug concentration for pharmacokinetic analysis (Shah et al., 1991).

New drug molecules are also produced to improve health outcomes. These drug molecules are always under scrutiny and undergo extensive *in vitro* and *in vivo* testing including pharmacokinetic evaluation. However, very few new molecules prove to be successful moiety and are marketed. Some of the existing products have low bioavailability and/or permeability. Formulation scientists utilize different techniques such as solid emulsifying drug delivery systems, liquisolid, and the formation of cocrystals to improve bioavailability. The most popular technique among these is nano-formulation, which has been successfully applied to improve the solubility and permeability of anticancer drugs. Nano-formulation can be used for targeted delivery systems thus achieving therapeutic goals by improving bioavailability (Atkinson, 2015).

When administered together, drugs can sometimes interact with each other and result in altered therapeutic outcomes. A study by Mushtaq et al. demonstrated that a single dose of clarithromycin significantly increased the bioavailability of voriconazole. The prolonged concomitant administration of these drugs may result in severe side effects of voriconazole because of an increase in Cmax and AUC and a decrease in clearance. Clinical trials are also designed to analyze pharmacokinetic drug-drug and drug-food interactions. Mostly these drugs are either metabolized by the same systems or/are substrates, inducers, or inhibitors of a particular enzyme. Similarly, if they utilize the same drug transporter system then the pharmacokinetic parameters can be altered resulting in a compromised outcome (Atkinson, 2015).

Pharmacokinetics and regulatory bodies such as the Food and Drug Administration (FDA) have also provided guidelines to the pharmaceutical industry on bioequivalence studies on drugs to which the biowaiver cannot be granted. Que et al. performed the bioequivalence study of extended-release of sitagliptin and metformin in healthy Chinese volunteers. The Cmax and AUC were within the acceptance limits and the new product was considered as bioequivalent to the branded product. Bioequivalence studies can be performed for monotherapies, drug combinations/fixed dose combinations, and controlled-release products. Pharmacokinetics of new fixed-dose combinations (drug combo) is also necessary because excipients and the APIs in the product may affect one another. In a single-dose study, the pharmacokinetic parameters of Maxigesic, a commercial product containing IBU 150 mg and paracetamol 500 mg per tablet, were not altered when taken either in fasted or fed state. No pharmacokinetic drug-drug interaction was observed between IBU and paracetamol by comparing the pharmacokinetics of IBU and paracetamol in FDC with that of monotherapy (Atkinson, 2015).

Physiologically based pharmacokinetic (PBPK) modeling and simulation studies using preclinical data can be used to predict the pharmacokinetic behavior of drugs in humans during various phases of drug discovery and development. These studies reduce the need for animal studies and have the potential to refine or even substitute clinical trials. PBPK modeling has become an integral tool in drug discovery and development in both pharmaceutical industries and academic platforms. Gradually it is being introduced for regulatory acceptance. The manuscript published by Zhang et al. shows that the PBPK model successfully simulates the tissue concentration that can be directly quantified.

In conclusion, this Research Topic highlights the importance of pharmacokinetics in drug development and also shows the significance of an appropriate analytical method in pharmacokinetic evaluation. Pharmacokinetics plays a key role in deciding the dosage regimen, and the dose can be adjusted in case there are unavoidable drug-drug interactions.
